# Expression of EpCAM in uveal melanoma

**DOI:** 10.1186/1475-2867-6-26

**Published:** 2006-11-24

**Authors:** Danilo N Odashiro, Alexandre N Odashiro, Patrícia R Pereira, Katyanne Godeiro, Emilia Antecka, Sebastian Di Cesare, Miguel N Burnier

**Affiliations:** 1 Henry C. Witelson Ocular Pathology Laboratory, McGill University, Montreal, QC, Canada; 2 Department of Ophthalmology, Federal University of São Paulo (UNIFESP), São Paulo, Brazil; 3 LAC-Pathology and Cytopathology Laboratory, Campo Grande, MS, Brazil; 4 Universidade para o Desenvolvimento do Estado e Região do Pantanal (UNIDERP) – Campo Grande, MS, Brazil

## Abstract

**Background:**

Uveal melanoma (UM) is the most common primary intraocular malignant tumor in adults, and nearly 40% of UM will develop metastasis that will ultimately lead to death. The Epithelial Cell Adhesion Molecule (EpCAM) is a type I transmembrane glycoprotein expressed by carcinomas of head and neck, ovary, colon, breast, kidney and lung. Recently, antibodies against EpCAM such as Edrecolomab and Catumaxomab were developed, and clinical trials with these antibodies have been used in several types of neoplasia. We studied the expression of EpCAM in UM.

**Methods:**

25 enucleated formalin-fixed, paraffin-embedded UM specimens were immunostained for EpCAM. Histopathological analysis of the specimens with regards to prognostic factors such as cell type, largest (linear) tumor dimension, number of mitotic figures, scleral invasion and tumor infiltrating lymphocytes were done.

**Results:**

None of them was positive for this EpCAM.

**Conclusion:**

In our report, UM did not express EpCAM. Therefore, it is not a helpful immunohistochemical marker to predict the behavior of UM. Further studies are needed to verify if EpCAM could also be related with prognosis and treatment of UM.

## Background

Uveal melanoma (UM) is the most common primary intraocular malignant tumor in adults and encompass nearly 85% of all ocular melanoma[1]. The worldwide incidence of UM is 5.3 to 10.9 cases per million population comprising about 4,25% of all melanomas[1]. Even with the progress of diagnosis and treatment methods, in the last 25 years the UM mortality is almost unaltered[1]. Nearly 40% of UM will develop metastasis that will ultimately lead to death; therefore, a better understanding of the cellular and molecular mechanisms of UM is essential in order to develop novel and specific drugs to prevent or treat UM metastasis. It has been postulated that the high malignancy of cutaneous[2] and uveal melanomas[3] could be connected with an increased cytokeratin expression, an epithelial cell marker.

Novel drugs that target cell surface antigens, signalling pathways, or critical effector molecules are in evidence in cancer research. We previously demonstrated that the majority of UM are positive for C-kit, a tyrosine kinase receptor, and UM cells impressively decreased the proliferation and invasion rates when exposed to imatinib mesylate, the C-kit inhibitor[4].

The Epithelial Cell Adhesion Molecule (EpCAM) discovered in the early 1980's is a type I transmembrane glycoprotein encoded by the ga733-2 gene on chromosome 4 (locus 4q). It is detected at the basolateral membrane of the majority of epithelial tissue such as simple, pseudoestratified and transitional epithelium. However, in mature squamous stratified epithelium and in hepatocytes, EpCAM is negative [5-10]. Prior studies reported that EpCAM express in a variety of epithelial neoplasias, like carcinomas of head and neck, ovary, colon, breast, kidney and lung[5,9,11]. EpCAM immunoreactivity was also found in squamous pre-malignant lesions[5].

Recently, antibodies against EpCAM such as Edrecolomab and Catumaxomab were developed. Clinical trials with these antibodies has been used in patients with Colon, Breast, Head and Neck, Ovary and Gastrointestinal carcinomas [11-15].

Until now, immunoreactivity against Ep-CAM has not been previously described in UM. The aim of this research is to study the expression of EpCAM in UM.

## Materials and methods

UM specimens obtained by enucleation between 1980 and 2004 were collected from the archives of the Henry C. Witelson Ocular Pathology Laboratory and Registry, McGill University, Montreal, Canada. Each specimen was formalin-fixed, paraffin-embedded and contained sufficient material for H&E and immunohistochemistry. Tumors presenting extensive necrosis that precluded an appropriate evaluation of histopathological features were excluded.

Histopathological analysis of the specimens with regards to prognostic factors such as cell type (modified Callender's classification) largest (linear) tumor dimension (LTD), number of mitotic figures in 40 high power fields (HPF) (400×), scleral invasion and tumor infiltrating lymphocytes (TIL) in 20 HPF were done. For the purpose of statistical analysis, tumors where classified as having a low mitotic rate (0–1 mitotic figures in 40 HPF) or a high mitotic rate (2 or more mitotic figures in 40 HPF). These parameters have been previously used in past studies. The presence of TIL was classified as low (< 200 lymphocytes in 20 HPF) or high (> 200 lymphocytes in 20 HPF) according to a previous publication.

Immunohistochemistry was performed using the monoclonal anti-EpCAM antibody VU-1D9 (ab11293 – abcam, Cambridge, MA, USA). The antibody was applied at a dilution of 1:70 for 18 h at 4°C, after 15 minutes in 10 nmol/l citrate buffer (pH 6.0) for antigen retrieval. Endogenous peroxidase was blocked using 0.3% hydrogen peroxidase diluted in methanol for 10 minutes. A standard avidin-biotin complex (ABC) technique using 3 amino-9 ethyl-carbazole was used for visualization. A case of colon adenocarcinoma was used as a positive control. Negative control sections were incubated with normal rabbit serum instead of the primary antibody.

After tissue processing, all cells that displayed distinct immunoreactivity were considered positive, regardless of intensity. Negative expression was determined by absence of the immunostain.

Statistical analysis was performed using a computer software (Statistical Package for the Social Sciences" version 11.5 (SSPS Inc., Chicago, IL, USA)). The categorical variables such as cell type (spindle or epithelioid), scleral invasion, mitosis (0–1 or ≥2 in 40 HPF) and TIL (0–200 or > 200 in 20 HPF) were analyzed. The chi-square test was used to assess statistical significance and a p value of less than 0.05 was considered significant.

## Results

The results are displayed in table 1.

**Table 1:** The histopathological features of the UM cases.

Case	Cell type	Scleral Invasion	TIL	Mitosis	LTD (mm)	EpCAM
1	M	N	L	H	13	Neg
2	M	N	L	L	7	Neg
3	M	N	L	H	12	Neg
4	M	N	L	H	9	Neg
5	E	Y	H	H	18	Neg
6	M	N	L	L	5	Neg
7	M	N	L	L	8	Neg
8	M	N	L	H	8	Neg
9	M	N	L	L	5	Neg
10	E	N	H	H	14	Neg
11	M	N	L	L	7	Neg
12	M	N	L	L	9	Neg
13	M	N	L	L	9	Neg
14	S	N	L	L	4	Neg
15	M	N	H	H	12	Neg
16	M	N	L	H	13	Neg
17	M	N	L	H	11	Neg
18	E	Y	H	H	16	Neg
19	M	N	L	L	7	Neg
20	M	N	L	H	11	Neg
21	E	N	H	H	13	Neg
22	M	N	L	L	6	Neg
23	S	N	L	L	4	Neg
24	E	N	H	H	14	Neg
25	M	N	L	H	12	Neg

TIL: tumor infiltrating lymphocytes; LTD: largest tumor dimension; M: mixed cell type; E: epithelioid cell tupe; S: spindle cell type; N: no; Y: yes; H: high; L: low; Neg: negative expression.

Twenty-nine cases of UM were retrieved, and four cases were excluded due to extensive tumor necrosis. The 25 UM specimens studied presented the following cell types: 18 mixed, 5 epithelioid and 2 spindle. Scleral invasion was observed in 8% of the studied cases (n = 2). The tumor infiltrating lymphocyte index was low in 76% of the cases (n = 19) and high in 24% of the remaining cases (n = 6). Approximately 56% of cases presented 2 or more mitotic figures in 40 HPF (n = 14) and the remaining had 1 or less (n = 11). The mean average of the LTD was 9,9 mm.

**Figure 1:**
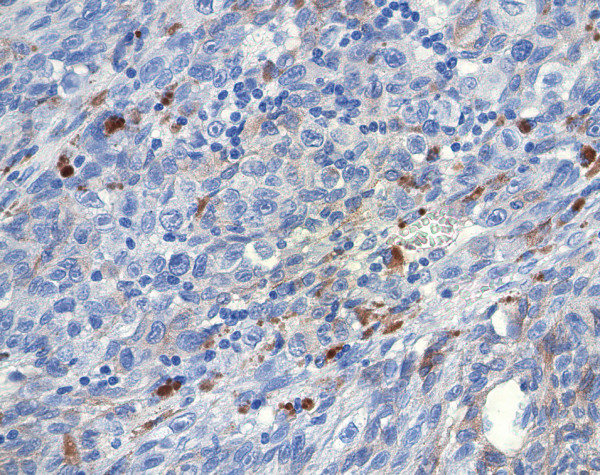
Epithelioid pigmented melanoma cells showing no expression to EpCam by immunohistochemistry.

All the 25 specimens demonstrated negative staining to EpCAM. There was no expression of this protein in any part of the enucleated specimens (figure 1).

## Discussion

EpCAM was described under several names originated from the respective monoclonal antibodies (KSA, ga733-2, 17-1a antigen, mh99, aua 1, moc31) [5-8]. It mediates Ca2+ independent homotypic cell-cell adhesions and was correlated with the preservation of cellular adhesion [5-10]. Previous studies demonstrated that the overexpression of EpCAM had a negative efect on E-cadherin mediated cell-cell adhesion and upregulated the proto-oncogene c-myc and cyclin A/E[16]. Based on these previous observations, EpCAM might be related with dediferentiation, proliferation and tumoral invasion[5,16].

EpCAM is expressed in several types of epithelial tumors[5,17]. EpCAM overexpression is correlated with poor disease free suvival in breast cancer[18], and loss of EpCAM expression in gastric adenocarcinoma has been reported to be associated with poor TNM staging prognosis[10], although inconsistent[19]. It is also detected weakly and occasionally in types of cancer other than carcinoma as fibrosarcoma, angiosarcoma, amd estesioneuroblastoma[17].

Some cases of cutaneous melanoma stains for epithelial markers[20], and most of them are metastatic lesions[21], meaning, more aggressive tumors. Also, UM cases that express cytokeratin proteins, a phenomenon that have been called interconverted phenotype, are correlated with poor prognosis[3]. In such cases, EpCAM, as an epithelial antigen, could be expected to be positive. Further studies of EpCAM with known cytokeratin positive UM tumors could confirm if there is any relationship between EpCAM and UM.

The reason of testing EpCAM in UM is the anti-EpCAM drugs avaiable to treat some malignant tumors. Edrecolomab is a monoclonal Anti-EpCAM antibody (Co17-1A antigen). In studies using Edrecolomab as adjuvant therapy, there was elimination of bone marrow micrometastases from Breast cancer[13]. Also, Edrecolomab has been tested in patients with colon cancer stages II and III, but without improvement in overall or disease-free survival, even when added with fluorouracil and folinic acid[14,22].

Catumaxomab is a bispecific trifunctional antibody (trAb) (anti-EpCAM × anti-CD3) and belongs to a new class of intact antibodies. The two binding sites of this antibody are against EpCAM positive tumor cells and T cells (CD3+). At the same time, this is mediated by the Fc-region, which binds to Fc3 – receptors I and III on accessory cells (macrophages, natural killer cells, dendritic cells). This results in higher tumor killing than the monoclonal antibodies mentioned[11,12,15,23]. Recently, there are promising results with Catumaxomab in treatment of malignant ascites associated with Ovarian cancer, Non-small cell Lung Cancer and Peritoneal Carcinomatosis due to Gastrointestinal Cancer[12,15,23].

We made an effort to detect EpCAM expression in 25 selected cases of uveal melanoma; however, none of them were positive, even in the UM epithelioid cell type.

## Conclusion

EpCAM is overexpressed in a variety of epithelial neoplasias. The development of adjuvant therapy targeted against EpCAM, in the future, may help increase patient survival in this set of patients. In our report, UM did not express EpCAM. Therefore, it is not a helpful immunohistochemical marker to predict the behavior of UM. Further studies are needed to verify if EpCAM could also be related with prognosis and treatment of UM.

## Authors' contributions

DNO – wrote the manuscript

ANO – revised the histopathological characteristics of the UM cases

PRP and KG – revised the expression of EpCam in the UM cases

EA and SDC – responsible for the immunostains

MB – revised the entire manuscript.
